# Dengue fever transmission between a construction site and its surrounding communities in China

**DOI:** 10.1186/s13071-020-04463-x

**Published:** 2021-01-06

**Authors:** Xingchun Liu, Meng Zhang, Qu Cheng, Yingtao Zhang, Guoqiang Ye, Xiqing Huang, Zeyu Zhao, Jia Rui, Qingqing Hu, Roger Frutos, Tianmu Chen, Tie Song, Min Kang

**Affiliations:** 1 State Key Laboratory of Molecular Vaccinology and Molecular Diagnostics, School of Public Health, Xiamen University, Xiamen, Fujian People’s Republic of China; 2 Guangdong Provincial Center for Disease Control and Prevention, Guangzhou, Guangdong People’s Republic of China; 3 Division of Environmental Health Sciences School of Public Health, University of California, Berkeley, CA 94720 USA; 4 Zhanjiang Municipal Center for Disease Control and Prevention, Zhanjiang, Guangdong People’s Republic of China; 5 Division of Public Health, School of Medicine, University of Utah, 201 Presidents Circle, Salt Lake City, UT 84112 USA; 6 University of Montpellier, Montpellier, France

**Keywords:** Dengue, Outbreak, Mathematical model, Construction site, Community

## Abstract

**Background:**

Due to an increase in mosquito habitats and the lack facilities to carry out basic mosquito control, construction sites in China are more likely to experience secondary dengue fever infection after importation of an initial infection, which may then increase the number of infections in the neighboring communities and the chance of community transmission. The aim of this study was to investigate how to effectively reduce the transmission of dengue fever at construction sites and the neighboring communities.

**Methods:**

The Susceptible-Exposed-Infectious/Asymptomatic-Recovered (SEIAR) model of human and SEI model of mosquitoes were developed to estimate the transmission of dengue virus between humans and mosquitoes within the construction site and within a neighboring community, as well between each of these. With the calibrated model, we further estimated the effectiveness of different intervention scenarios targeting at reducing the transmissibility at different locations (i.e. construction sites and community) with the total attack rate (TAR) and the duration of the outbreak (DO).

**Results:**

A total of 102 construction site-related and 131 community-related cases of dengue fever were reported in our area of study. Without intervention, the number of cases related to the construction site and the community rose to 156 (TAR: 31.25%) and 10,796 (TAR: 21.59%), respectively. When the transmission route from mosquitoes to humans in the community was cut off, the number of community cases decreased to a minimum of 33 compared with other simulated scenarios (TAR: 0.068%, DO: 60 days). If the transmission route from infectious mosquitoes in the community and that from the construction site to susceptible people on the site were cut off at the same time, the number of cases on the construction site dropped to a minimum of 74 (TAR: 14.88%, DO: 66 days).

**Conclusions:**

To control the outbreak of dengue fever effectively on both the construction site and in the community, interventions needed to be made both within the community and from the community to the construction site. If interventions only took place within the construction site, the number of cases on the construction site would not be reduced. Also, interventions implemented only within the construction site or between the construction site and the community would not lead to a reduction in the number of cases in the community.
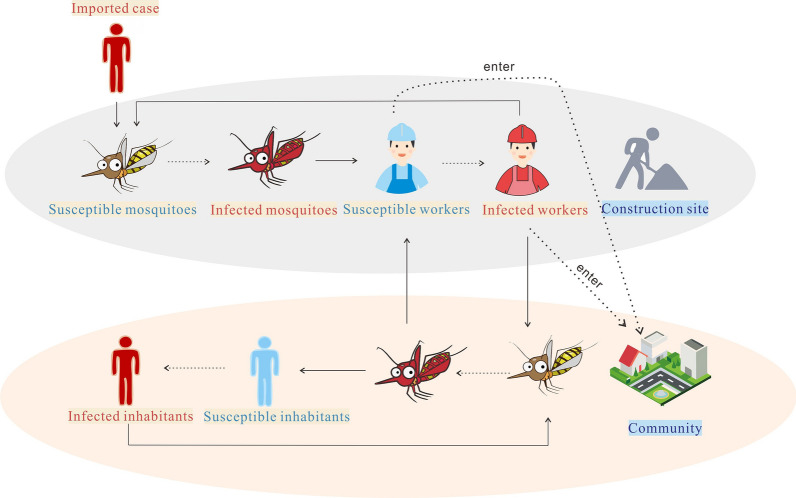

## Introduction

Dengue fever (DF) is one of the most rapidly spreading mosquito-borne diseases in the world. It is caused by four different serotypes of dengue virus (DENV 1–4) transmitted by female *Aedes* mosquitoes [1, 2]. The incidence rate has increased by 30-fold over the past 50 years, with more than 100 countries currently faced with the threat of DF. According to an estimation made in 2013, the total number of people infected worldwide reaches 390 million each year, which places a heavy healthcare burden on governments and individuals [3–5]. The incidence rate of DF in China ranges from 0.0091 to 3.4581 per 100,000 people, with a total of 52,749 new dengue cases reported between 2009 and 2014 [6]. The rapidly increasing rate of urbanization has provided the *Aedes* mosquito with many new breeding sites, and this increase in breeding sites has become important risk factor for DF. For example, containers, such as waste containers, tires and water storage tanks, are major sites of *Aedes* mosquito breeding [7], with increased population density and mobility also facilitating the propagation of the virus [2, 8]. These environmental conditions and the lack of basic mosquito control facilities have made construction sites in high-risk areas hotspots for outbreaks. Moreover, following importation of an initial case, construction sites are more likely to be affected by secondary infections, which will increase the number of infections being transmitted to the neighboring communities and, thereby, the chance of community transmission. A study published in 2018 suggested that the overall case burden of construction site-associated clusters was significantly high through three case studies of large construction site-associated DF clusters during 2013–2016 [9].

Guangdong is a sub-tropical province in southern China, characterized by a rapid urbanization process and dense population. From 1949 to 1977, there were no reports of DF cases in China [10]. The first outbreak of DF was reported in 1978 in Foshan City, Guangdong Province, and this province has had the highest infection rate of DF in Mainland China since then [11]. Between 2008 and 2018, more than 85% of all domestic dengue cases in China occurred in Guangdong province [12], with the most serious DF outbreak in China in the past 20 years reported in Guangdong province in June 2014, with more than 45,000 people infected [10, 13]. However, the Chinese government has not yet developed an effective vaccination to prevent the disease [14, 15]. The dengue serotypes detected in Guangzhou were mainly DENV 1 and DENV 2 [16]. *Aedes* *albopictus* mosquitoes, which are densely distributed throughout Guangdong province, are considered to be the main vector. Due to the absence of specific treatment and effective vaccination for DF, vector control remains the only strategy to prevent the disease [17].

**Fig. 1:**
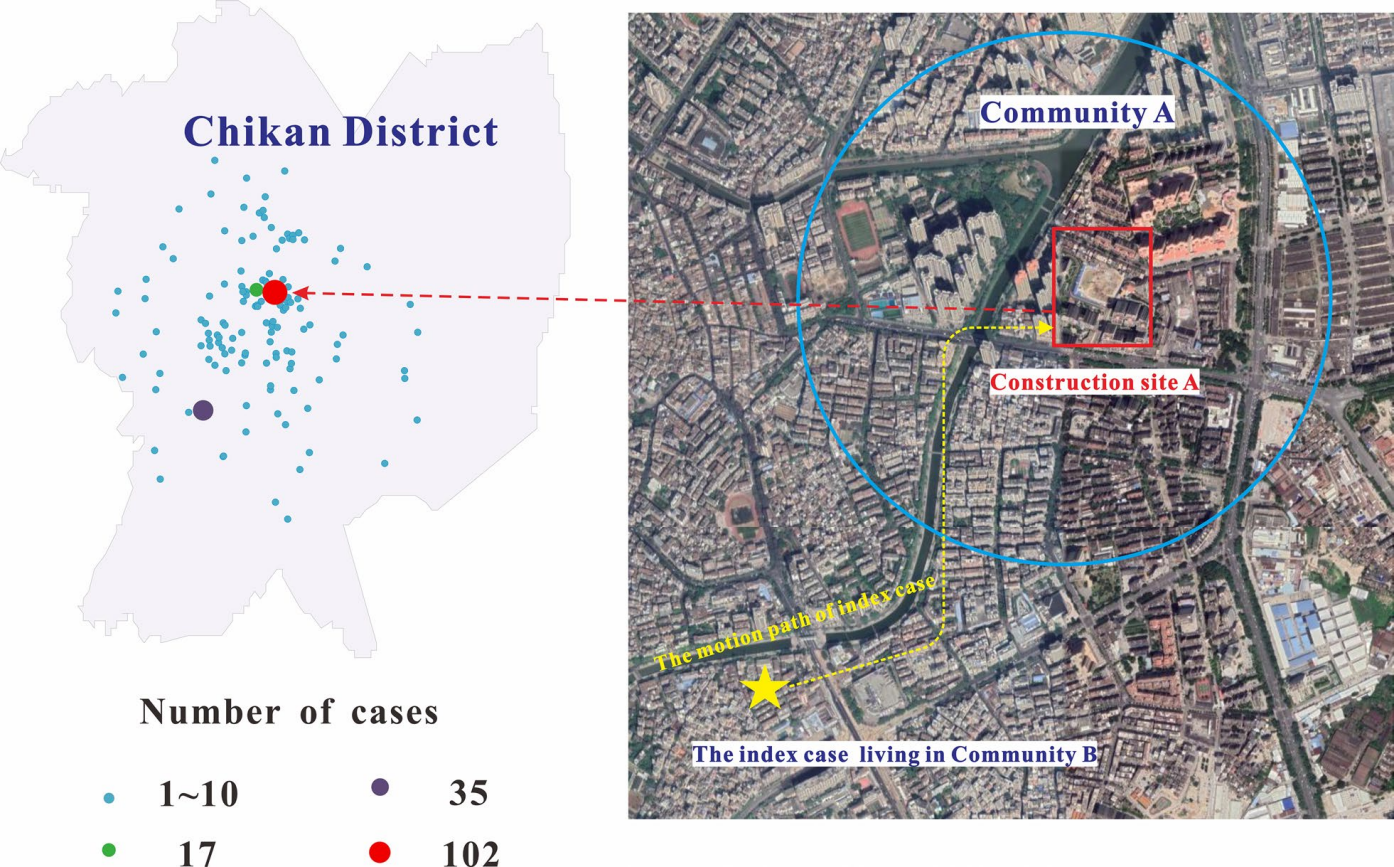
Distribution of dengue cases in Chikan District in 2018 and remote sensing images of Community A and the index case. Filled red circle represents construction site A located in the middle of Chikan District

In 2018, a serious DF outbreak occurred in Zhanjiang Prefecture, Guangdong Province. From the first indigenous DF case reported on July 19 to the last case on October 28, a total of 467 cases were reported, which was 7.9-fold higher than the number of DF cases reported in Zhanjiang Prefecture in 2017 (59 cases). Most of these cases occurred in Community A of Chikan District (Zhanjiang Prefecture), accounting for 50% (233 cases) of all reported cases. The transmission spread from construction site A (102 cases) to the surrounding residential areas (131 cases). The distribution of DF cases in Chikan District and the remote sensing images of Community A are shown in Fig. 1.

Due to the scope and severity of this outbreak, the main aim of this research is to investigate the transmissibility of DF on the construction site and into the surrounding communities and to determine the practical significance of implementing interventions in different regions.

Prior to this study, there have been many studies on DF using mathematical models [18–21]. Esteva and Vargas established a mathematical model to analyze the role of vertical transmission and mechanical transmission due to interrupted feeding in the dynamics of dengue disease [18]. Favier and Schmit established a model that underlined the critical influence of the spatial structure of population scale, and stressed that contact heterogeneity must be considered to reproduce the epidemiological curve [19]. Li et al. presented a temperature-driven coupled entomological–epidemiological model and assessed the role of seasonal vector dynamics and infection importation in driving dengue outbreaks [20]. Hartley et al. introduced seasonally varying parameters into a mathematical model of the transmission dynamics of dengue viruses in a stepwise manner [22]. However, there has never been a model that involves the interaction between a construction site and the neighboring community in terms of DF transmission. Mathematical models have also been applied for other mosquito-borne diseases. Ghosh et al. developed a deterministic model governed by a system of non-linear differential equations to assess the effects of recurrent malaria; global asymptotic dynamics of the autonomous model and the non-autonomous model with time-dependent control strategies were applied to make the best plan for malaria control [23]. A Zika virus transmission model with three nonlinear forces of infection was formulated by Olaniyi et al. and the optimal control theory of Pontryagin’s maximum principle was used to identify the best measures to control the spread of Zika virus disease [24].

In the present study, we developed an Susceptible-Exposed-Infectious/Asymptomatic-Removed (SEIAR) model for the host and an Susceptible-Exposed-Infectious (SEI) model for the vector, with the aim to simulate the transmission dynamics of dengue virus and evaluate the effectiveness of different intervention measures [21]. This is the first model to investigate the transmissibility of DF on a construction site and in the neighboring community and to analyze their interactions.

## Methods

### Study site

Zhanjiang Prefecture is a prefecture-level city located in Guangdong Province. Chikan District (110°20′–110°21′E, 21°14′–21°19′N) is the central area of Zhanjiang Prefecture. Chikan District has a subtropical maritime monsoon climate, with a long summer and short winter and a mean yearly rainfall of 1596 mm. The rainy season is accompanied by high temperature. There are about 300,000 persons residing in Chikan District, which occupies 79 km^2^. The study area includes a community (Community A) and a nearby construction site A. Community A is located in northeast-central Chikan District, and has a dense population of about 50,000 people who are highly mobile, both factors which facilitate the spread of DF. The construction site A is located at the center of Community A, and has a workforce of about 500 permanent persons, most of whom work at the construction site during the day and travel to the surrounding communities after work where they live. *Aedes* *albopictus* is the only vector species in this region for DF [25].

### Disease data

In the outbreak studied here, 447 cases of infection were locally acquired, of which 15 cases were imported from other provinces of Mainland China and five cases were imported from abroad. We define an indigenous case as an individual who contracted DF and who had not left the province (current address) within 14 days before the onset of disease. An imported case of DF was defined as an infected patient who had been to a dengue endemic region within 14 days before the onset of illness [26].

All DF cases were identified following the diagnostic criteria (WS216-2008) announced by the National Health Commission (formerly named as the Ministry of Health) of the People's Republic of China [27], as follows:

1. Clinically diagnosed case: a suspected case with leucopenia or thrombocytopenia; or a suspected case whose serum tested positive for immunoglobulin (Ig)G or IgM.
2. Laboratory-confirmed case: a suspected case whose serum tested positive for DENV RNA by real-time PCR; or isolation of the DENV from the blood, tissue or cerebrospinal fluid of a patient with acute infection; or an IgG titer in the recovery period fourfold higher than that in the acute period.

Data on all clinically diagnosed and laboratory-confirmed cases relating to this DF outbreak were obtained from the field epidemiological investigation conducted by Guangdong Center for Disease Control and Prevention (CDC). Demographic information included age, gender, address and occupation of the cases. The study period was defined as beginning on 17 August 17 (date when the putative index case was reported) and ending on 2 October (date when the last case in Community A was reported). We considered the case reported on August 17 in Zhanjiang Prefecture as the index case in our study area, given the facts that: (1) the extrinsic incubation period of DF is about 10 days [21] and the time interval between this case and the first case in our study area on the construction site A on August 27 falls into the typical range of the serial interval of dengue; (2) this case lives in Community B, Chikan District, which is < 1 km away from the construction site A; (3) the case visited the construction site several times before August 27. The residential location and the activity path of the index case are shown in Fig. 1. The local CDC introduced interventions on 12 September, including case isolation, environmental cleaning, spraying of insecticides and surveillance of Breteau Index,, which may have changed the transmissibility of the disease. Therefore, we used data collected before September 12 in our model to simulate transmissibility without intervention.

### Model structure

**Fig. 2:**
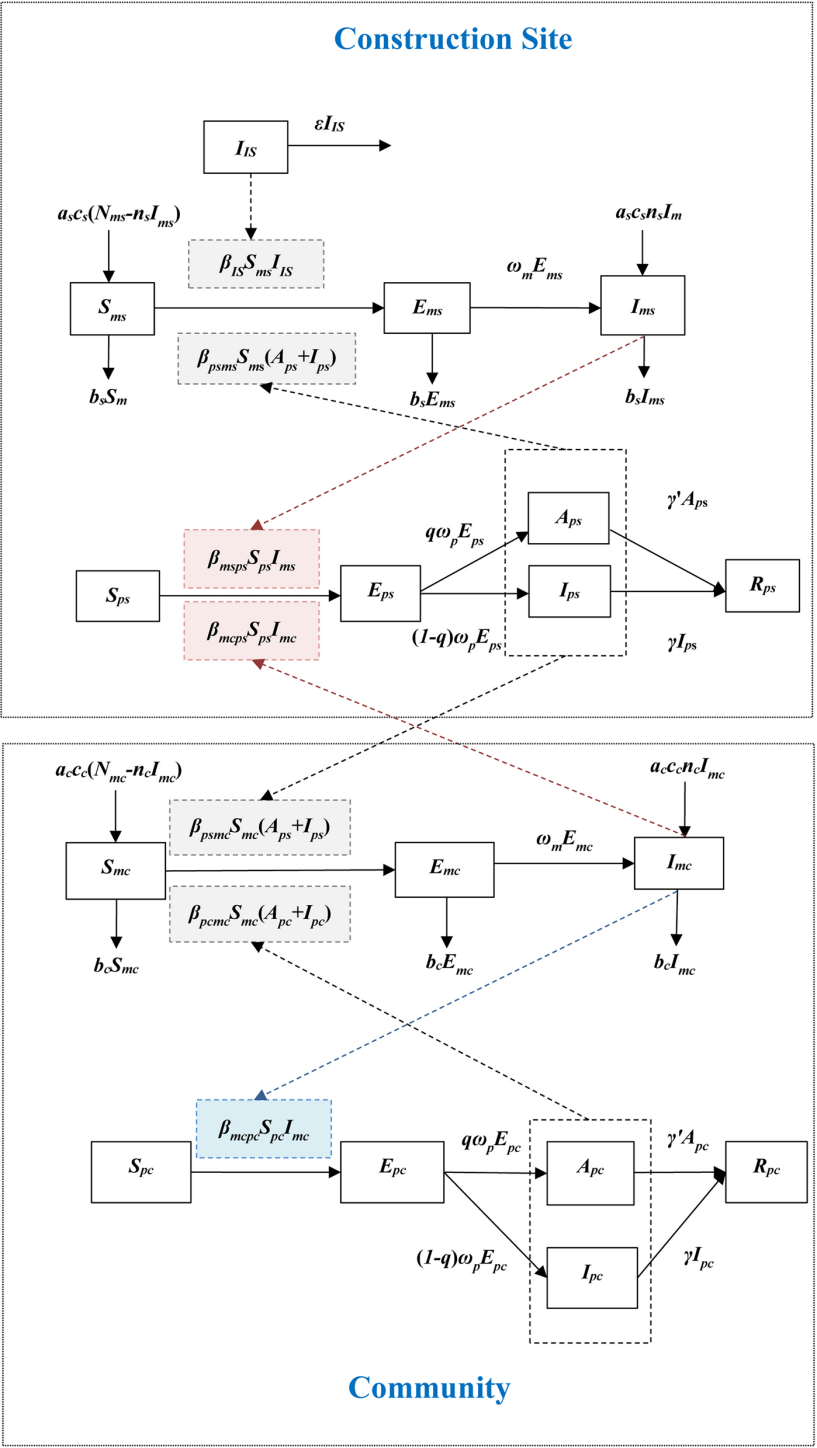
Flowchart of the development of the dengue transmission model. Parts in red represent two ways to control the number of dengue cases on the construction site to a minimum, the part in blue represents the way to control the number of dengue cases in the community to a minimum. For an explanation of the terminology, refer to “Model structure” section

We developed a model to simulate the transmission of dengue virus within the residential areas, on construction site A located in Community A, and between the residential areas and construction site A. The model is based on our previous studies in which it was used to study the dynamic transmission of mosquito-borne diseases [21, 25]. The model structure is shown in Fig. 2. In the model, individuals on the construction site can be divided into the following five elements: *S*_ps_, susceptible; *E*_ps_, exposed; *I*_ps_, infectious; *A*_ps_, asymptomatic; *R*_ps_, removed, with the subscript ‘ps’ representing people on the construction site. *I*_IS_ represents cases imported into the construction site. Mosquitoes on the construction site can be divided into the following three elements: *S*_ms_, susceptible; *E*_ms_, exposed; *I*_ms_, infectious, where the subscript ‘ms’ represents mosquitoes on the construction site. *N*_ms_ represents the sum of the three mosquito elements. Each element of the SEIAR–SEI model in the residential areas of the community is represented by the same symbol as that for the site and has the same meaning. We simplified the residential areas of the community as community. The human elements in community were divided into: *S*_pc_, susceptible; *E*_pc_, exposed; *I*_pc_, infectious; *A*_pc_, asymptomatic; *R*_pc_, removed, with the subscript ‘pc’ representing people in the community. Mosquitoes in the community can be divided into the following three elements: *S*_mc_, susceptible; *E*_mc_ exposed; *I*_mc_, infectious, with the subscript ‘mc’ representing mosquitoes in the community.

The model is based on the following assumptions or facts:

1. The imported case transmitted the virus to a construction worker, who further transmitted it to other construction workers and community residents. The first case occurred in the residential areas around construction site A on September 8, which was 13 days later than the first case on construction site A. The initial value of the number of imported cases *I*_IS_ was set to 1.
2. The susceptible individuals become either asymptomatic or symptomatic after a latent period or incubation period. The parameter *q* represents the proportion of asymptomatic individuals, so the (1 −* q*)*E* exposed individuals would eventually enter the *I* compartment, while the other *qE* exposed individuals would end up in the *A* compartment.
3. Humans recover with immunity, while infected mosquitoes are infectious until death.
4. According to the field epidemiological investigation conducted by Guangdong CDC, construction workers stayed on construction site A during the day, with most returning to their respective homes in Community A after work, while residents of the community who were not construction workers were not allowed to enter the construction site due to safety considerations.
5. Mosquitoes on the construction site stay only there and cannot fly to the community areas, and* vice versa*, since studies have shown that the mean flight distance of *Ae.* *albopictus* rarely exceed 75 m [28, 29]. Therefore, the movement of construction workers is the only way to transmit the virus between the construction site and the community.

The mathematical model is described by the following ordinary differential equations (ODE):

$$\frac{{{\text{d}}I_{{{\text{Is}}}} }}{{{\text{d}}t}} = - \varepsilon I_{{{\text{Is}}}}$$

$$\frac{{{\text{d}}S_{{{\text{ms}}}} }}{{{\text{d}}t}} = \, a_{s} c_{s} \left( {N_{{{\text{ms}}}} - n_{s} I_{{{\text{ms}}}} } \right) - \beta_{{{\text{Is}}}} S_{{{\text{ms}}}} I_{{{\text{Is}}}} - \beta_{{{\text{ps}}\;{\text{ms}}}} S_{{{\text{ms}}}} \left( {A_{{{\text{ps}}}} + I_{{{\text{ps}}}} } \right) - b_{s} S_{{{\text{ms}}}}$$

$$\frac{{{\text{d}}E_{{{\text{ms}}}} }}{{{\text{d}}t}} = \beta_{{{\text{Is}}}} S_{{{\text{ms}}}} I_{{{\text{Is}}}} + \beta_{{{\text{psms}}}} S_{{{\text{ms}}}} \left( {A_{{{\text{ps}}}} + I_{{{\text{ps}}}} } \right) - \left( {b_{s} + \omega_{m} } \right)E_{{{\text{ms}}}}$$

$$\frac{{{\text{d}}I_{{{\text{ms}}}} }}{{{\text{d}}t}} = a_{s} c_{s} n_{s} I_{{{\text{ms}}}} + \omega_{m} E_{{{\text{ms}}}} - b_{s} I_{{{\text{ms}}}}$$

$$N_{{{\text{ms}}}} = S_{{{\text{ms}}}} + E_{{{\text{ms}}}} + I_{{{\text{ms}}}}$$

$$\frac{{{\text{d}}S_{{{\text{ps}}}} }}{{{\text{d}}t}} = - \beta_{{{\text{msps}}}} S_{{{\text{ps}}}} I_{{{\text{ms}}}} - \beta_{{{\text{mcps}}}} S_{{{\text{ps}}}} I_{{{\text{mc}}}}$$

$$\frac{{{\text{d}}E_{{{\text{ps}}}} }}{{{\text{d}}t}} = \beta_{{{\text{msps}}}} S_{{{\text{ps}}}} I_{{{\text{ms}}}} + \beta_{{{\text{mcps}}}} S_{{{\text{ps}}}} I_{{{\text{mc}}}} - \omega_{p} E_{{{\text{ps}}}}$$

$$\frac{{{\text{d}}I_{{{\text{ps}}}} }}{{{\text{d}}t}} = \left( {1 - q} \right)\omega_{p} E_{{{\text{ps}}}} - \gamma I_{{{\text{ps}}}}$$

$$\frac{{{\text{d}}A_{{{\text{ps}}}} }}{{{\text{d}}t}} = q\omega_{p} E_{{{\text{ps}}}} - \gamma^{\prime}A_{{{\text{ps}}}}$$

$$\frac{{{\text{d}}R_{{{\text{ps}}}} }}{{{\text{d}}t}} = \gamma I_{{{\text{ps}}}} + \gamma^{\prime}A_{{{\text{ps}}}}$$

$$\frac{{{\text{d}}S_{{{\text{mc}}}} }}{{{\text{d}}t}} = \, a_{c} c_{c} \left( {N_{{{\text{mc}}}} - n_{c} I_{{{\text{mc}}}} } \right) - \beta_{{{\text{pcmc}}}} S_{{{\text{mc}}}} \left( {A_{{{\text{pc}}}} + I_{{{\text{pc}}}} } \right) - \beta_{{{\text{psmc}}}} S_{{{\text{mc}}}} \left( {A_{{{\text{ps}}}} + I_{{{\text{ps}}}} } \right) - b_{c} S_{{{\text{mc}}}}$$

$$\frac{{{\text{d}}E_{{{\text{mc}}}} }}{{{\text{d}}t}} = \, \beta_{{{\text{pcmc}}}} S_{{{\text{mc}}}} \left( {A_{{{\text{pc}}}} + I_{{{\text{pc}}}} } \right) + \beta_{{{\text{psmc}}}} S_{{{\text{mc}}}} \left( {A_{{{\text{ps}}}} + I_{{{\text{ps}}}} } \right) - \left( {b_{c} + \omega_{m} } \right)E_{{{\text{mc}}}}$$

$$\frac{{{\text{d}}I_{{{\text{mc}}}} }}{{{\text{d}}t}} = a_{c} c_{c} n_{c} I_{{{\text{mc}}}} + \omega_{m} E_{{{\text{mc}}}} - b_{c} I_{{{\text{mc}}}}$$

$$N_{{{\text{mc}}}} = S_{{{\text{mc}}}} + E_{{{\text{mc}}}} + I_{{{\text{mc}}}}$$

$$\frac{{{\text{d}}S_{{{\text{pc}}}} }}{{{\text{d}}t}} = - \beta_{{{\text{mcpc}}}} S_{{{\text{pc}}}} I_{{{\text{mc}}}}$$

$$\frac{{{\text{d}}E_{{{\text{pc}}}} }}{{{\text{d}}t}} = \beta_{{{\text{mcpc}}}} S_{{{\text{pc}}}} I_{{{\text{mc}}}} - \omega_{p} E_{{{\text{pc}}}}$$

$$\frac{{{\text{d}}I_{{{\text{pc}}}} }}{{{\text{d}}t}} = \left( {1 - q} \right)\omega_{p} E_{{{\text{pc}}}} - \gamma I_{{{\text{pc}}}}$$

$$\frac{{{\text{d}}A_{{{\text{pc}}}} }}{{{\text{d}}t}} = q\omega_{p} E_{{{\text{pc}}}} - \gamma^{\prime}A_{{{\text{pc}}}}$$

$$\frac{{{\text{d}}R_{{{\text{pc}}}} }}{{{\text{d}}t}} = \gamma I_{{{\text{pc}}}} + \gamma^{\prime}A_{{{\text{pc}}}}$$

**Fig. 3:**
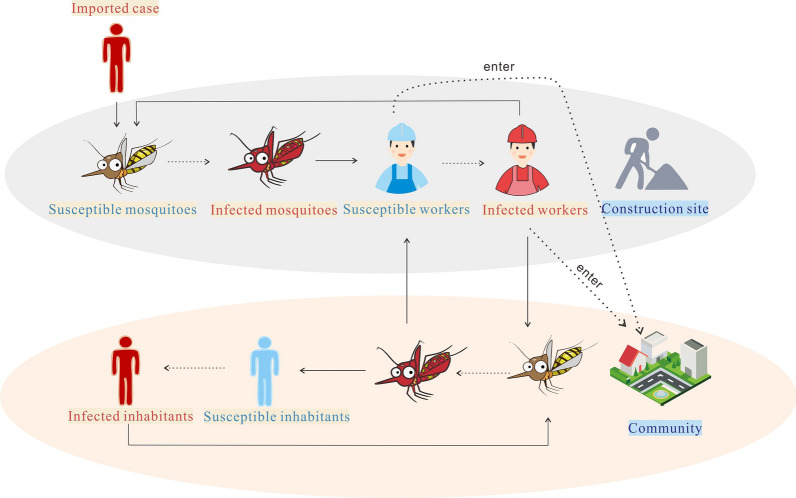
The transmission process of dengue virus between the construction site and the neighboring community

A simplified transmission process diagram was drawn to simulate the transmission of DENV between the construction site and the community (Fig. 3).

### Parameter estimation

We set all other parameters according to the literature (Table 1) and only fitted the values for *β*_IS,_ *β*_psms,_ *β*_msps,_ *β*_mcps,_ *β*_psmc,_ *β*_pcmc_ and *β*_mcpc._ Since these parameters were fitted to data before the start of interventions on September 12, we denoted them as *β*_unc_. The incubation period of DENV in humans usually spans a 4- to 8-day period; thus the value of 6 days was selected as the average value, with *ω*_*p*_ = 0.1667 per day [30]. The extrinsic incubation period, i.e. the time interval between mosquito infection and when its bite become infectious [31], is 8–12 days; thus we selected 10 days in the simulation, with *ω*_*m*_ = 0.1000 per day. The ratio of symptomatic to asymptomatic infection of DF is 2.2:1 [32]; thus *q* = 0.6875. The infectious period is 3–14 days [31]; we assumed it to be 7 days in the model; thus *γ* *=* *γ'* *=* *ε* *=* 0.1429 per day. The birth rate and death rate of mosquitoes were set to be *a*_*s*_ *=* *a*_*c*_ *=* *b*_*s*_ *=* *b*_*c*_ *=* 0.0714, in accordance with a previous study [21]. The vertical infection rates of individual positive families of DENV-1 range from 1.4 to 17.4% [33]; for our model we assumed it to be 10.0%, based on previous work [21]; thus *n*_*s*_ *=* *n*_*c*_ = 0.1000. As the abundance of *Ae.* *albopictus* varies with seasons and regions [34], we introduced two seasonal parameters, *c*_*s*_ and *c*_*c*_, and used a trigonometric function to simulate these according to a previous study [35]. The calculation of parameters *c*_*s*_ and *c*_*c*_ were as follows:

$$c_{s} = c_{c} = \cos \left[ {\frac{{2\pi \left( {t - \tau } \right)}}{T}} \right]$$

where *τ* and *T* refer to simulation delay of the initial time in the whole season and the time span of the season cycle, respectively.

**Table 1:** Parameter definitions and values

Parameter	Description	Unit	Value	Range	Method
*β*_IS_	Transmission relative rate from imported human case to mosquitoes on the construction site	1	1.67742 × 10^−5^	≥ 0	Curve fitting
*β*_psms_	Transmission relative rate from people on the construction site to mosquitoes on the construction site	1	0.00200626	≥ 0	Curve fitting
*β*_msps_	Transmission relative rate from mosquitoes on the construction site to humans on the construction site	1	0.00101487	≥ 0	Curve fitting
*β*_mcps_	Transmission relative rate from mosquitoes in the community to people on the construction site	1	0.00301141	≥ 0	Curve fitting
*β*_psmc_	Transmission relative rate from people on the construction site to mosquitoes in the community	1	3.3702×10^−15^	≥ 0	Curve fitting
*β*_pcmc_	Transmission relative rate from people in the community to mosquitoes in the community	1	0.00523777	≥ 0	Curve fitting
*β*_mcpc_	Transmission relative rate from mosquitoes in the community to people in the community	1	1.12805 × 10^−5^	≥ 0	Curve fitting
*ω*_*m*_	Incubation relative rate of mosquito infection	Day^−1^	0.1000	0.0833–0.1250	[31]
*ω*_*p*_	Incubation relative rate of human infection	Day^−1^	0.1667	0.1250–0.2500	[30]
*q*	Proportion of human asymptomatic infection	1	0.6875	0–1	[32]
*γ*	Removed relative rate of infectious individuals	Day^−1^	0.1429	0.0714–0.3333	[31]
*γ*′	Removed relative rate of asymptomatic individuals	Day^−1^	0.1429	0.0714–0.3333	[31]
*ε*	Removed relative rate of imported individuals	Day^−1^	0.1429	0.0714–0.3333	[31]
*a*_*s*_	Daily birth rate of mosquitoes on the construction site	Day^−1^	0.0714	0.0200–0.2500	[21]
*a*_*c*_	Daily birth rate of mosquitoes in the community	Day^−1^	0.0714	0.0200–0.2500	[21]
*b*_*s*_	Daily death rate of mosquitoes on the construction site	Day^−1^	0.0714	0.0200–0.2500	[21]
*b*_*c*_	Daily death rate of mosquitoes in the community	Day^−1^	0.0714	0.0200–0.2500	[21]
*τ*	Simulation delay in the initial time in the whole season day	Day	242	≥ 0	Analysis on the reported data
*T*	Duration of the daily cycle	Day	365	≥ 0	Analysis on the reported data
*c*_*s*_*,* *c*_*c*_	Seasonality parameter of the mosquito population	1	See text	0–1	Curve fitting
*n*_*s*_*,* *n*_*c*_	Proportion of transovarial transmission	1	0.1000	0.0140–0.1740	[21]

According to the reported data, the illness onset date of the infection source was 17 August and the peak of the disease spanned September. To calculate the seasonal parameters *c*_*s*_ and *c*_*c*_, we simulated the seasonality of the vector population dynamics with a cycle of 12 months (where *T* = 365) for accuracy and to achieve a better effect for model simulation, thus *τ* = 242.

### Evaluating the effectiveness of different intervention strategies

We assessed the effectiveness of different intervention scenarios, with the aim to reduce the transmissibility of DENV between different populations and mosquito subgroups at different places (e.g. between infected construction workers and susceptible mosquitoes on the construction site, and between infected mosquitoes and susceptible community residents in the community). We examined the scenarios of setting one to all seven *β*_unc_ to zero by assuming that the intervention is perfect and the targeted *β*_unc_ can be reduced to zero. We set the time of intervention as the same as the actual situation, which means that after the disease develops at the original speed for a period of time, the targeted *β*_unc_ will only become zero after 12 September.

1. Only one coefficient *β* was controlled to be zero. Firstly, we made *β*_IS_ = 0 or *β*_psms_ = 0 or *β*_msps_ = 0 or *β*_mcps_ = 0 or *β*_psmc_ = 0 or *β*_pcmc_ = 0 or *β*_mcpc_ = 0, respectively. At the same time, we kept the other six coefficients *β* unchanged.
2. Two coefficients *β* were controlled to be zero. Then we combined these seven coefficients *β*_unc_ in pairs, so that two of them were equal to zero; we kept the other five coefficients *β* unchanged at the same time. There were a total of 21 combinations.
3. Three coefficients *β* were controlled to be zero. Three of these seven coefficients were set to zero, while the others remained unchanged. There were a total of 35 combinations.
4. Four coefficients *β* were controlled to be zero. We made four out of these seven coefficients equal to zero, while the others remained unchanged. There were a total of 35 combinations.
5. Five coefficients *β* were controlled to be zero. We set five out of these seven coefficients to zero, while the others remained unchanged. There were a total of 20 combinations.
6. Six coefficients *β* were controlled to be zero. We made six of these seven coefficients equal to zero, while the others remained unchanged. There were a total of 7 combinations.
7. Seven coefficients *β* were controlled to be zero. We made all seven coefficients equal to zero.

The specific meaning of controlling *β*_unc_ to be zero is explained as follows:

- *β*_IS_ = 0 means cutting off the transmission route from the imported case to susceptible mosquitoes on the construction site.
- *β*_psms_ = 0 means cutting off the transmission route from infectious and symptomatic people to susceptible mosquitoes on the construction site.
- *β*_msps_ = 0 means cutting off the transmission route from infectious mosquitoes to susceptible people on the construction site.
- *β*_mcps_ = 0 means cutting off the transmission route from infectious mosquitoes in the community to susceptible people on the construction site.
- *β*_psmc_ = 0 means cutting off the transmission route from infectious and symptomatic people on the construction site to susceptible mosquitoes inside the community.
- *β*_pcmc_ = 0 means cutting off the transmission route from infectious and symptomatic people to susceptible mosquitoes inside the community.
- *β*_mcpc_ = 0 means cutting off the transmission route from infectious mosquitoes to susceptible people inside the community.

### Indicators for assessing the effectiveness of interventions

The effectiveness of the interventions is represented by absolute effectiveness (AE) and relative effectiveness (RE), which are calculated as:

$${\text{AE}}_{i} = {\text{TAR}}_{i} - {\text{TAR}}_{{{\text{baseline}}}}$$

$${\text{RE}}_{i} = \left( {{\text{TAR}}_{i} - {\text{TAR}}_{{{\text{baseline}}}} } \right)/{\text{TAR}}_{i} \times 100\%$$

where TAR_*i*_ represents the total attack rate, defined as the proportion of population being infected (only symptomatic) during the simulation period, TAR_baseline_ represents the TAR when *β* are kept at the fitted values using data before 12 September.

From the simulation results, we obtained the number of susceptible people and infected people at different times. We first considered the number of susceptible people when the number of infected persons was 1, which means the disease was effectively controlled at this time. We then subtracted the number of susceptible people at this time from the total number of people at the start on the construction site or in the community to obtain the total number of infected people, considering in this process that there were not only asymptomatic but also symptomatic individuals. Finally, we obtained the TAR by dividing the total number of people infected by the proportion of symptomatic individuals. Duration of outbreak (DO) represents the number of days calculated from the beginning to the date when the number of infected people reaches the value of one.

### Simulation method

We used Berkeley Madonna ver. 8.3.18 (developed by Robert Macey and George Oster of the University of California at Berkeley, CA, USA) for parameter fitting and model simulation. The goodness-of-fitting was assessed by least root-mean-square error between simulated and observed number of new cases per day between 17 August and 12 September. The coefficient of determination (*R*^2^) was assessed using SPSS ver. 13.0 (IBM Corp., Armonk, NY, USA) to quantify the significance of the fit.

## Results

### Epidemiological characteristics of the outbreak

**Fig. 4:**
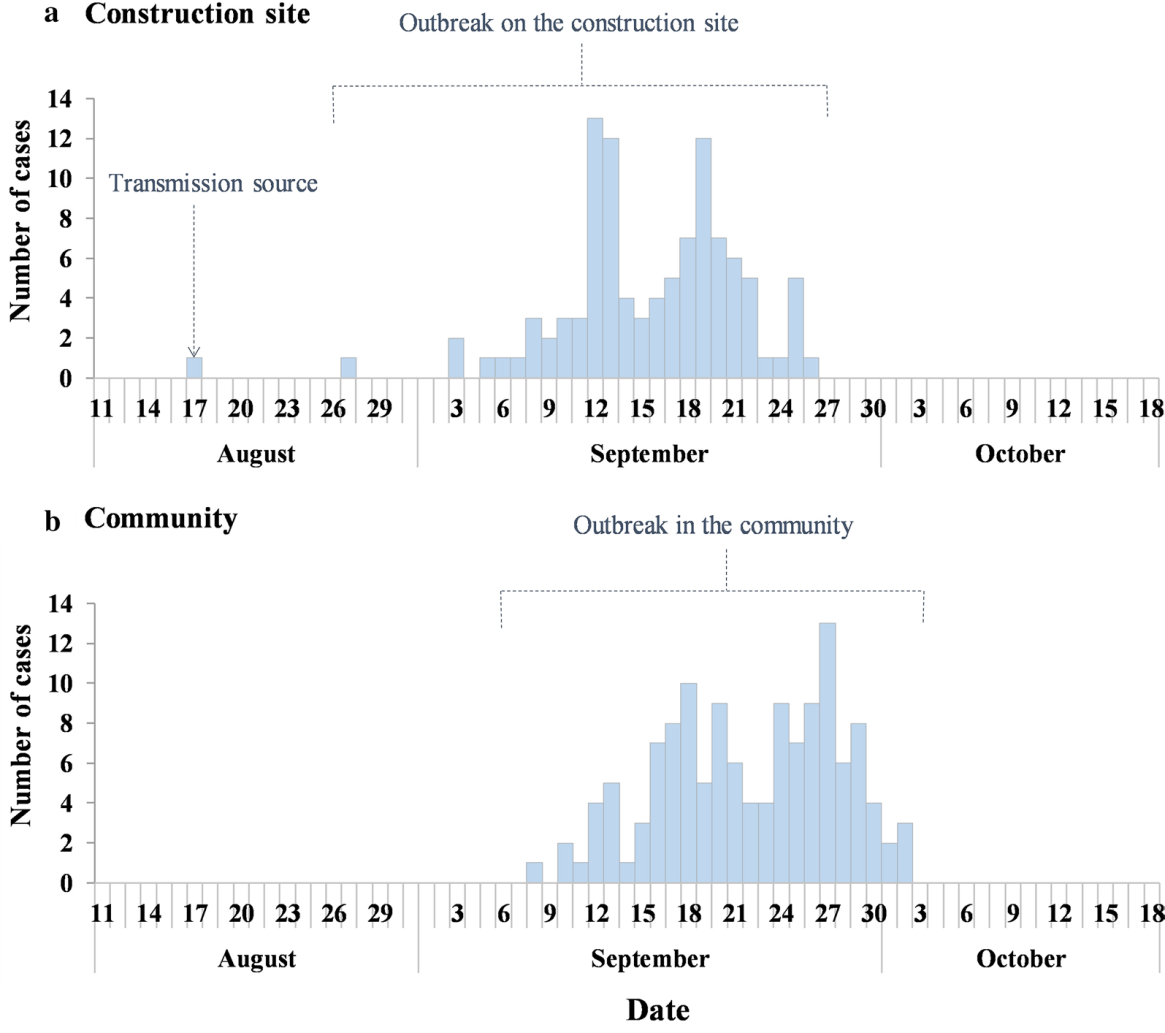
Reported cases of dengue fever on the construction site A and in the surrounding community in Zhanjiang Prefecture, P.R. China, in 2018

A total of 467 cases of DF were reported in Zhanjiang Prefecture in 2018, and the first indigenous case was reported in Chikan District on 29 August. The number of new cases on the construction site and in the community per day are shown in Fig. 4. The first indigenous case was reported on the construction site A within Community A in Chikan District on 27 August, then it spread within the construction site. In early September, DF began to spread to the surrounding communities, causing the outbreak. The number of indigenous cases began to increase on 3 September, and the number of new cases reached 13 on 12 September, following which date Zhanjiang Municipal CDC began to implement intervention measures. The outbreak on the construction site ended in late September with a total of 102 cases while the community outbreak continued to spread until 2 October with a cumulative number 131 cases. The outbreak source was identified as a 30-year old woman living in the community close to the construction site who developed symptoms on 17 August.

### Results of curve fitting

**Fig. 5:**
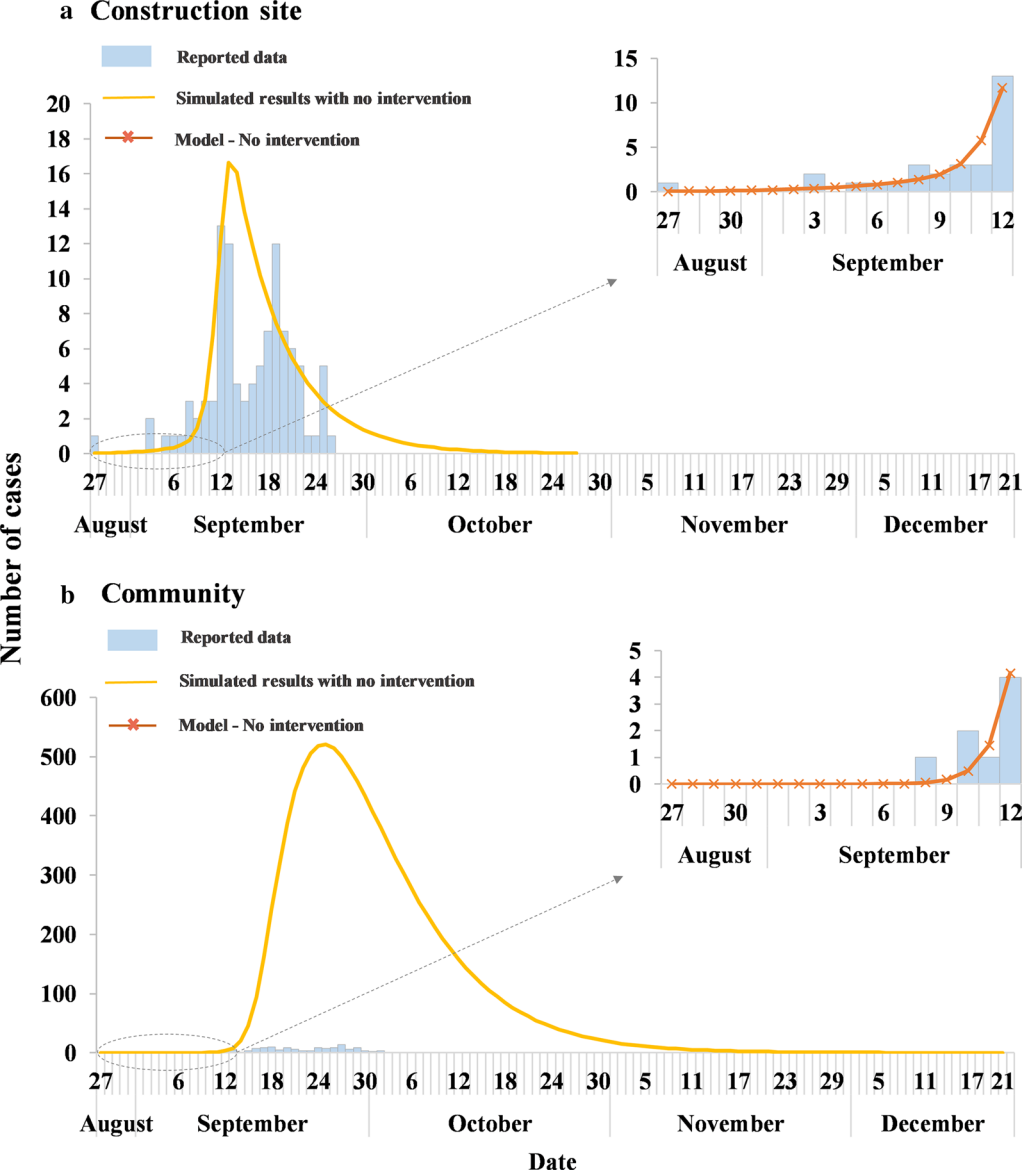
Curve fitting of reported data and Susceptible-Exposed-Infectious/Asymptomatic-Recovered (SEIAR) models without intervention and simulated epidemic curve with no intervention on the construction site (**a**) and in the neighboring community (**b**)

The model fitting results are shown in Fig. 5. The values of *R*^2^ were 0.829 (*P* < 0.001) and 0.878 (*P* < 0.001) for the construction site and the community, respectively. Therefore, the optimal values of *β*_IS_, *β*_psms_, *β*_msps_, *β*_mcps_, *β*_psmc_, *β*_pcmc_, *β*_mcpc_ are generated by curve fitting (summarized in Table 1).

### Simulation for the baseline scenario

We used the fitted *β*_unc_ to simulate the baseline scenario when no intervention measures were applied. The simulation results indicated that in the absence of any intervention, the number of cases on the construction site would reach 156, yielding a TAR of 31.25% (95% confidence interval [CI] 27.18–35.31%) and a DO of 73 days, while in the community, the number of cases would reach up to 10,796, yielding a TAR of 21.59% (95% CI 21.23–21.95%) and a DO of 127 days (Fig. 5). The AE and RE of the outbreak control strategy implemented by Zhanjiang Municipal CDC on the construction site were 10.65 and 34.08%, respectively, and the AE and RE of the community were 21.33 and 98.79%, respectively.

### Effectiveness of different intervention strategies

The effectiveness of different intervention strategies were investigated by setting each *β*_unc_ or the combination of multiple *β*_unc_ to 0. The first scenario has only one coefficient *β* controlled to be zero: when we controlled *β*_IS_ = 0 or *β*_psms_ = 0 or *β*_msps_ = 0 or *β*_psmc_ = 0, we found that these measures were not efficient, and the number of cases was the same as the uncontrolled data. When *β*_mcps_ = 0, that is, when the transmission route from infectious mosquitoes in the community to susceptible mosquitoes on the construction site was cut off, the number of cases on the construction site would reach 129, yielding a TAR of 25.81% (95% CI 21.97–26.65%) and a DO of 85 days, with no effect in the community. When *β*_pcmc_ = 0, that is, when the transmission route from infectious and symptomatic people to susceptible people inside the community was cut off, the number of cases in the community would reach 2794, yielding a TAR of 5.60% (95% CI 5.40–5.80%) and a DO of 145 days, with no effect on the construction site. When *β*_mcpc_ = 0, that is, when the transmission route from infectious mosquitoes to susceptible people inside the community was cut off, the number of cases in the community would go down to 33, yielding a TAR of 0.068% (95% CI 0.05%–0.09%) and a DO of 60 days, with no effect on the construction site.

The second scenario is that two coefficient *β* are controlled to be zero. When we controlled *β*_psms_ = 0 and *β*_mcps_ = 0 at the same time, the number of cases on the construction site would drop to 103. When we controlled *β*_msps_ = 0 and *β*_mcps_ = 0 at the same time, that is, when the transmission route from infectious mosquitoes in the community and on the construction site to susceptible people on the construction site were cut off at the same time, the number of cases on the construction site would drop to 74.

The following scenarios are three or more *β*_unc_ changed to be zero. After more than 100 simulations, we found that even if we controlled *β*_IS_, *β*_psms_, *β*_msps_, *β*_mcps_, *β*_psmc_, *β*_pcmc_, *β*_mcpc_ to zero at the same time, the number of cases on the construction site would not be less than 74 and the number of cases in the community would not be less than 33. Detailed data of these results are shown in Additional file 1: Tables S1–S6.

## Discussion

Guangdong Province, Mainland China, suffers from serious outbreaks of DF. This province has a dense and highly mobile population and a large number of immigrants. In our study, the outbreak first occurred on the construction site, followed by outbreak in the community. This sequence indicates that once the transmission route is established, the virus may circulate between the construction site and the surrounding areas.

We developed an ODE model and applied the model to examine the effectiveness of different intervention scenarios aimed at reducing the transmissibility between different human and mosquito subpopulations at different places (i.e. the construction site and the nearby community). The coefficient of determination showed a high good-of-fitness of our models to the reported data, which indicated that this model is well fitted to similar situations characterized by urbanization where construction sites and communities are in close proximity.

Our simulation results showed that after an index case was introduced to the construction site but there were no intervention measures, the epidemic started earlier on the construction site compared to the community, even though the first indigenous case moved back and forth between the construction site and the community on a daily basis. Given the high mosquito abundance on the construction site, the virus could be more easily to be spread there than in the community. As the total case counts on the construction site increased, the probability of local transmission in the community also increased. Detecting cases in the high-risk areas could be used as an early warning signal to apply local interventions. We found that when the intervention consisted of only controlling transmission inside the construction site, it would have no any impact on reducing the transmission of the epidemic on the site. However, the number of cases on the construction site would reach a minimum when we cut off the transmission route from infectious mosquitoes in the community and the construction site to susceptible people on the construction site at the same time. In other words, only by controlling mosquitoes in the community, would the number of DF cases on the construction site be reduced.

Areas characterized by poor hygiene, (high) humidity, presence of multiple water-holding containers and a dense population are ideal breeding places for *Aedes* mosquitoes [36]. Construction sites satisfy all of these requirements. In terms of spreading infection, *Aedes* mosquitoes are more likely to bite during the day, with biting activity increasing within 2 h after sunrise and a few hours after sunset, which is when workers are working outside and therefore easier targets in terms of infection. Also, workers generally work the whole day without protective measures or wearing light clothes, which may facilitate becoming infected with mosquito-borne diseases [37]. In addition, such factors as densely populated construction sites, high labor intensity and high temperature are significantly related to the increase in the number of DF cases [38]. Moreover, due to the high level of urbanization in China, the number of construction sites are increasing steadily, together with a large population of construction workers and massive mobility [9]. Once external cases are imported, DF may be prevalent on construction sites. Therefore, construction sites should be regularly inspected, and the places where mosquitoes breed and workers are prevalent should be checked to detect early cases. However, just controlling the *β* between mosquitoes and people inside the construction site is not sufficient, possibly due to the fact that the transfer of the virus by humans to naive populations of mosquitoes in the communities must also be considered. *Aedes* mosquitoes involved in dengue transmission do not fly more than 75 m from their breeding sites; therefore, the transmission of DF over relatively longers distancs necessarily involves humans, either directly as an infected source or indirectly by transporting mosquitoes.

When focusing on the community, our study demonstrated that the number of cases in the community would increase to an extremely high level within a short period of time without any intervention and that controlling the transmission route on the construction site or between the construction site and the community would not decrease the number of dengue cases of the community. We found that the number of community cases would be significantly reduced only when we controlled the transmission routes within the community, and the outbreak in the community would be significantly controlled when we cut off the transmission route from infectious mosquitoes to susceptible people inside the community. Furthermore, controlling the transmission within the construction site alone only had a marginal impact on the number of cases in the community in our study, since the population size of of construction workers is much smaller compared to the number of community inhabitants. Therefore more attention should be paid to the interaction between people and vector inside the community to control DF at the community level.

Community participation is considered to be the key to controlling endemic diseases, especially in the case of DF, as the activity of insects is closely related to lifestyle and housing in urban areas [39]. Mosquitoes in the community will not only cause outbreaks of disease within the community, but they also affect the surrounding construction sites, causing a wider spread of DF. In the past few decades, the main method to prevent and control DF has been to reduce mosquito breeding sites and control their density [8]. However, this method often fails to achieve good results due to the lack of active community participation. It is necessary for communities to plan anti-mosquito actions in an organized manner, such as by propagating the importance of DF prevention, regularly inspecting houses, killing insects found on various containers, especially water storage containers [40], and controlling adult mosquito density.

Of note, our study has the following limitations. First, we did not take the death toll from the disease into account. Even though the fatality rate of DF is extremely low, when the outbreak is more serious and lasts longer, fatality rate may have a slight impact on the simulated data. Second, we did not simulate multiple countermeasures in this outbreak, such as case isolation, adult vector control and larvae control, thus the specific effect of different measures was not explored. Third, because of the complex model structure and because DF is transmitted through a vector rather than simply from person to person, we could not calculate *R*_0_ (the basic reproduction number) to assess the transmissibility of this disease with or without intervention. In future work, we should explore a feasible method to solve this problem.

## Conclusions

When the infection source of dengue is introduced to a construction site, the epidemic will first break out on the site. Without intervention, DF could subsequently break out rapidly into the densely populated communities around the construction site. The ODE models can be useful to simulate the outbreak of DF and to predict the transmissibility of the disease. Controlling the transmission route within the construction site or between the construction site and the community was found not to curb the outbreak on both the construction site and in the community. Cutting off the transmission routes and properly controlling vectors in communities should be recommended as primary measures to control this disease.

## Supplementary information

**Additional file 1: Table S1.** Simulated results of scenario 1. **Table S2.** Simulated results of scenario 2. **Table S3.** Simulated results of scenario 3. **Table S4.** Simulated results of scenario 4. **Table S5.** Simulated results of scenario 5. **Table S6.** Simulated results of scenario 6 and scenario 7.

## Data Availability

Data supporting the conclusions of this article are included within the article.

## Abbreviations

CDC: Center for Disease Control and Prevention
DF: Dengue fever
DO: Duration of the outbreak
ODE: Ordinary differential equation
*R*_0_: Basic reproduction number
*R*^*2*^: Coefficient of determination
SEIAR: Susceptible–exposed–symptomatic–asymptomatic–recovered/removed (model)
TAR: Total attack rate
CI: Confidence interval

## References

[1] Lai S, Huang Z, Zhou H, Anders KL, Perkins TA, Yin W (2015). The changing epidemiology of dengue in China, 1990–2014: a descriptive analysis of 25 years of nationwide surveillance data. BMC Med..

[2] Bhatt S, Gething PW, Brady OJ, Messina JP, Farlow AW, Moyes CL (2013). The global distribution and burden of dengue. Nature.

[3] Guzman MG, Harris E (2015). Dengue. Lancet.

[4] Lee HS, Nguyen-Viet H, Nam VS, Lee M, Won S, Duc PP (2017). Seasonal patterns of dengue fever and associated climate factors in 4 provinces in Vietnam from 1994 to 2013. BMC Infect Dis..

[5] Li Q, Cao W, Ren H, Ji Z, Jiang H (2018). Spatiotemporal responses of dengue fever transmission to the road network in an urban area. Acta Trop..

[6] Chen B, Liu QY (2015). Dengue fever in China. Lancet.

[7] Van Benthem BH, Vanwambeke SO, Khantikul N, Burghoorn-Maas C, Panart K, Oskam L (2005). Spatial patterns of and risk factors for seropositivity for dengue infection. Am J Trop Med Hyg..

[8] Hermann LL, Gupta SB, Manoff SB, Kalayanarooj S, Gibbons RV, Coller BA (2015). Advances in the understanding, management, and prevention of dengue. J Clin Virol..

[9] Liang S, Hapuarachchi HC, Rajarethinam J, Koo C, Tang CS, Chong CS (2018). Construction sites as an important driver of dengue transmission: implications for disease control. BMC Infect Dis..

[10] Wu JY, Lun ZR, James AA, Chen XG (2010). Dengue Fever in mainland China. Am J Trop Med Hyg..

[11] Fan J, Lin H, Wang C, Bai L, Yang S, Chu C (2014). Identifying the high-risk areas and associated meteorological factors of dengue transmission in Guangdong Province, China from 2005 to 2011. Epidemiol Infect..

[12] Zhao S, Musa SS, Meng J, Qin J, He D (2020). The long-term changing dynamics of dengue infectivity in Guangdong, China, from 2008–2018: a modelling analysis. Trans R Soc Trop Med Hyg..

[13] Huang L, Luo X, Shao J, Yan H, Qiu Y, Ke P (2016). Epidemiology and characteristics of the dengue outbreak in Guangdong, Southern China, in 2014. Eur J Clin Microbiol Infect Dis..

[14] Liu J, Tian X, Deng Y, Du Z, Liang T, Hao Y (2019). Risk ractors associated with dengue virus Infection in Guangdong Province: a community-based case–control study. Int J Environ Res Public Health..

[15] Guo Y, Song Z, Luo L, Wang Q, Zhou G, Yang D (2018). Molecular evidence for new sympatric cryptic species of *Aedes**albopictus* (Diptera: Culicidae) in China: A new threat from *Aedes**albopictus* subgroup?. Parasites Vectors..

[16] Sang S, Chen B, Wu H, Yang Z, Di B, Wang L (2015). Dengue is still an imported disease in China: a case study in Guangzhou. Infect Genet Evol..

[17] San Martin JL, Brathwaite O, Zambrano B, Solorzano JO, Bouckenooghe A, Dayan GH (2010). The epidemiology of dengue in the Americas over the last three decades: a worrisome reality. Am J Trop Med Hyg..

[18] Esteva L, Vargas C (2000). Influence of vertical and mechanical transmission on the dynamics of dengue disease. Math Biosci..

[19] Favier C, Schmit D, Muller-Graf CD, Cazelles B, Degallier N, Mondet B (2005). Influence of spatial heterogeneity on an emerging infectious disease: the case of dengue epidemics. Proc Biol Sci..

[20] Li MT, Sun GQ, Yakob L, Zhu HP, Jin Z, Zhang WY (2016). The Driving Force for 2014 Dengue Outbreak in Guangdong, China. PLoS ONE.

[21] Yi B, Chen Y, Ma X, Rui J, Cui JA, Wang H (2019). Incidence dynamics and investigation of key interventions in a dengue outbreak in Ningbo City, China. PLoS Negl Trop Dis..

[22] Bartley LM, Donnelly CA, Garnett GP (2002). The seasonal pattern of dengue in endemic areas: mathematical models of mechanisms. Trans R Soc Trop Med Hyg..

[23] Ghosh M, Olaniyi S, Obabiyi O (2020). Mathematical analysis of reinfection and relapse in malaria dynamics. Appl Math Comput..

[24] Olaniyi SJAM (2018). Dynamics of Zika virus model with nonlinear incidence and optimal control strategies. Appl Math Inf Sci.

[25] Cheng Q, Jing Q, Spear RC, Marshall JM, Yang Z, Gong P (2016). Climate and the timing of imported cases as determinants of the dengue outbreak in Guangzhou, 2014: evidence from a mathematical model. PLoS Negl Trop Dis.

[26] Sun J, Zhang H, Tan Q, Zhou H, Guan D, Zhang X (2018). The epidemiological characteristics and molecular phylogeny of the dengue virus in Guangdong, China, 2015. Sci Rep..

[27] Peng HJ, Lai HB, Zhang QL, Xu BY, Zhang H, Liu WH (2012). A local outbreak of dengue caused by an imported case in Dongguan China. BMC Public Health..

[28] Honorio NA, Silva Wda C, Leite PJ, Goncalves JM, Lounibos LP, Lourenco-de-Oliveira R (2003). Dispersal of Aedes aegypti and Aedes albopictus (Diptera: Culicidae) in an urban endemic dengue area in the State of Rio de Janeiro. Brazil. Mem Inst Oswaldo Cruz..

[29] Verdonschot PFM, Besse-Lototskaya AA (2014). Flight distance of mosquitoes (Culicidae): A metadata analysis to support the management of barrier zones around rewetted and newly constructed wetlands. Limnologica..

[30] Chan M, Johansson MA (2012). The incubation periods of Dengue viruses. PLoS ONE.

[31] Andraud M, Hens N, Marais C, Beutels P (2012). Dynamic epidemiological models for dengue transmission: a systematic review of structural approaches. PLoS ONE.

[32] Wang T, Wang M, Shu B, Chen XQ, Luo L, Wang JY (2015). Evaluation of inapparent dengue infections during an outbreak in Southern China. PLoS Negl Trop Dis..

[33] Grunnill M, Boots M (2016). How important is vertical transmission of dengue viruses by mosquitoes (Diptera: Culicidae)?. J Med Entomol..

[34] Torres C, Barguil S, Melgarejo M, Olarte A (2014). Fuzzy model identification of dengue epidemic in Colombia based on multiresolution analysis. Artif Intell Med..

[35] Yang HM, Macoris ML, Galvani KC, Andrighetti MT, Wanderley DM (2009). Assessing the effects of temperature on the population of Aedes aegypti, the vector of dengue. Epidemiol Infect..

[36] Atique S, Chan TC, Chen CC, Hsu CY, Iqtidar S, Louis VR (2018). Investigating spatio-temporal distribution and diffusion patterns of the dengue outbreak in Swat Pakistan. J Infect Public Health..

[37] Sang S, Yin W, Bi P, Zhang H, Wang C, Liu X (2014). Predicting local dengue transmission in Guangzhou, China, through the influence of imported cases, mosquito density and climate variability. PLoS ONE.

[38] Burattini MN, Chen M, Chow A, Coutinho FA, Goh KT, Lopez LF (2008). Modelling the control strategies against dengue in Singapore. Epidemiol Infect..

[39] Caprara A, Lima JW, Peixoto AC, Motta CM, Nobre JM, Sommerfeld J (2015). Entomological impact and social participation in dengue control: a cluster randomized trial in Fortaleza, Brazil. Trans R Soc Trop Med Hyg..

[40] Wai KT, Htun PT, Oo T, Myint H, Lin Z, Kroeger A (2012). Community-centred eco-bio-social approach to control dengue vectors: an intervention study from Myanmar. Pathog Glob Health..

